# MicroRNAs and Developmental Robustness: A New Layer Is Revealed

**DOI:** 10.1371/journal.pbio.1000397

**Published:** 2010-06-15

**Authors:** Noam Shomron

**Affiliations:** Department of Cell and Developmental Biology, Sackler Faculty of Medicine, Tel Aviv University, Tel Aviv, Israel

## Abstract

MicroRNAs provide a new layer of regulation to ensure that a developmental program of programmed cell death yields a reproducible outcome in spite of perturbations to the system.

It does not happen often that an entirely novel gene regulatory mechanism is revealed. The discovery of microRNAs (miRNAs) is one such finding that revolutionized our understanding of cellular events and of the intricacy of developmental processes [1],[2]. These small (∼22 nucleotide), single-stranded RNA molecules act through binding in a sequence-specific manner to the 3′UTR of mRNA targets, an event that leads to facilitated mRNA degradation or translational inhibition [3]. With a very short recognition sequence determining its specificity for target mRNAs, each miRNA can potentially regulate hundreds of transcripts, though in many cases the physiological effects of miRNA targeting can be attributed to its binding to one major mRNA transcript. Each genome encodes hundreds of potential miRNA genes, and their expression is often widespread within the tissues of an organism. Although miRNAs lurked undetected until only relatively recently, it is now well established that miRNAs play an essential role in the regulation of many cellular processes [4].

The concept of robustness during the development of an organism or tissue refers to the ability of a developmental program to yield a reproducible outcome in spite of perturbations to the system, whether they are genetic (e.g., gene duplication), epigenetic (e.g., gene expression levels), or environmental (e.g., stress) in nature. One of the basic building blocks of a developmental program is mRNA synthesis, which takes place in intense and random bursts [5]. The multitude and amplitude of fluctuations in gene expression, leading to significant variation between cells, can derail developmental programs that rely on strict levels of regulatory factors. To deal with this, cells have evolved molecular mechanisms that ensure developmental robustness in the face of such intrinsically random fluctuations [6].

miRNA-mediated regulation has been proposed as one such mechanism for conferring robustness throughout development [7],[8]. In *Drosophila*, the Carthew laboratory recently provided the first strong experimental evidence to demonstrate that a miRNA, *miR-7*, acts to buffer developmental regulatory networks against perturbation [9]. Interestingly, the critical function of *miR-7* is evident only when the system is subjected to environmental stress in the form of temperature instability. Thus, this study supported the notion that miRNAs can contribute to developmental stability under conditions of environmental instability. The prime role of miRNAs as the guardian of mRNA levels, however, was not shown for development under normal physiological conditions.

The *Drosophila* eye is an ideal model for exploring the processes of morphogenesis. Composed of thousands of cells of various different cell types, each compound eye is in fact a simple hexagonal array of stereotyped clusters of cells called ommatidia. The interommatidial lattice also includes sense organs called interommatidial bristles, which are mechanosensory hair cells believed to protect the eye surface. During organ formation an orchestrated series of steps involving activation of cell proliferation, differentiation, and migration takes place. An additional critical part of morphogenesis in the eye—as in many developing neural systems—is programmed cell death, or apoptosis, which is used to remove excess cells after the correct organ pattern has been established. Excess interommatidial cells in the immature organ are removed by two waves of apoptosis during early pupal stages to produce the array of ommatidia found in the adult eye [10]. An important question is how the developing eye decides how many and which cells will survive and which will be removed during this apoptotic phase.

Several different miRNAs have been shown to regulate apoptosis in *Drosophila*. Brennecke et al. [11] demonstrated that *bantam* miRNA functions during tissue growth. Both *miR-14* and *miR-8* exhibit anti-apoptotic characteristics [12],[13], whereas *miR-2* family members regulate the pro-apoptotic genes *reaper*, *grim*, and *sickle* [14]. Although implicated previously in the regulation of apoptosis, none of the mutants that affect the members of this family have yet shown any role in developmental fine-tuning through apoptotic trimming of excess cells.

In an elegant study in this issue of *PLoS Biology*, the Cohen laboratory [15] describe a conserved miRNA family*—miR-263a/b*—that is expressed in the mechanosensory cells of the developing *Drosophila* eye and that plays a role in protecting fly bristles from apoptosis during the pruning event that forms the mature organ (Figure 1). The researchers show that in *miR-263a/b* deletion mutants' loss of bristles appears to be sporadic and excessive. The activity of these anti-apoptotic miRNAs appears to be to ensure that a sufficient number of interommatidial bristles are protected during the developmentally programmed wave of cell death that prunes the tissue in order to produce the correct pattern of the adult retina. Based on the observation that flies deficient for these miRNAs exhibit random bristle loss, the Cohen laboratory propose that these miRNAs play a protective role against excess apoptosis and thereby support robustness in the development of this complex organ. Interestingly, *miR-263a/b* are members of a conserved family of miRNAs that are expressed in peripheral sense organs across the animal kingdom and therefore may play a similar role in ensuring developmental robustness in other organisms.

**Figure 1:**
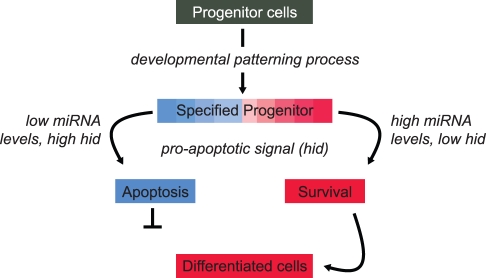
Robustness in the form of an anti-apoptotic effect is seen during a developmental patterning process. The fate of specified progenitor cells is determined by the levels of protective miRNAs expressed in those cells. Red and blue colors (and shades in between) represent high and low levels, respectively, of *miR-263a/b*, which targets the pro-apoptotic gene *hid*.

The exact stoichiometric relationship between a miRNA and its target that is required to confer functional regulation in vivo remains an open question. In the Cohen lab's study [15], it seems that a “more-than-needed” level is present, given that almost full restoration of the wild-type phenotype is seen in mutant flies grown under controlled conditions upon reintroduction of a transgene expressing only a fraction of the wild-type level of the miRNA. One possible explanation for this is that this regulatory system might be optimized for a level of perturbation encountered in the wild that is not encountered in the lab environment. In order to buffer against substantial natural fluctuation in target gene expression in the natural environment, a large amount of this miRNA might be required. Another possibility is that loss of even a small number of interommatidial bristles reduces fitness in the wild, so the system has evolved excess regulatory capacity to ensure robustness. Whichever the underlying reason, the features of this regulatory system imply that miRNAs enable a considerable buffering capacity to ensure that the process of interommatidial bristle formation is stable in an unstable developmental environment.

Each miRNA is capable of targeting a large number of genes [16]–[18]. Although in vitro assays show that many of these candidate target genes can be regulated by the miRNA, rigorous in vivo work is required to identify the relevant target genes in a physiologically relevant system. A question that arises from miRNA studies is how many miRNA targets are relevant to the miRNA's role in a particular system. Apparently, there are examples of both promiscuous and highly specific target regulation. Some reports indicate that the role of the miRNA may be to down-regulate many genes at the same time (e.g., [19],[20]). Others have identified distinct phenotypes for one mutant miRNA in different tissues, each linked to regulation of distinct, single targets (e.g., [13],[21]). In the Cohen study, it is clearly presented that the *hid* transcript is the biologically dominant and relevant target. Having said that, it is hard to rule out that other targets, or a combinatorial contribution of several of them, might be involved. Future work that is able to sort miRNAs according to these two modes of regulation, and to determine whether a miRNA can shift from one target-specific regulation to a wide-spread mode of target regulation based on the cellular context, certainly will be of interest as we continue to unravel the details of miRNA-mediated regulatory systems.

This research from the Cohen lab is particularly valuable because of the difficulty in studying in vivo a process that by definition maintains stability. Through studies such as this, however, researchers are beginning to explain the mechanisms by which the effects of noise—stochastic variation in gene expression—are minimized in complex tissues. In future studies, it would be of interest to understand what cascade of events regulates the intricate expression of *miR-263a/b* in bristle progenitors and not in neighboring cells, whether this protective effect occurs elsewhere during morphogenesis, and, given the high degree of conservation of *miRNA-263a/b* sequence and of their expression in sensory organs across phyla, whether this regulatory mechanism exists in other systems, including perhaps those associated with mammalian development and related diseases (for e.g., see [22]–[24]).

In summary, Cohen's work elegantly demonstrates, in a complex tissue, a role for miRNAs in conferring robustness of a unique and different sort, ensuring the survival of sense organ cells during developmental tissue pruning. This finding provides a valuable experimental validation of the concept of miRNA-mediated developmental robustness and adds yet another layer in our understanding of cellular events governed by miRNAs.
